# Defining gut mycobiota for wild animals: a need for caution in assigning authentic resident fungal taxa

**DOI:** 10.1186/s42523-021-00134-z

**Published:** 2021-10-28

**Authors:** Anton Lavrinienko, Tiffany Scholier, Scott T. Bates, Andrew N. Miller, Phillip C. Watts

**Affiliations:** 1 Department of Biological and Environmental Science, University of Jyväskylä, 40014 Jyväskylä, Finland; 2 Department of Biological Sciences, Purdue University Northwest, Westville, IN 46391 USA; 3 University of Illinois Urbana-Champaign, Illinois Natural History Survey, 1816 South Oak Street, Champaign, IL 61820-6970 USA

**Keywords:** Amplicon sequencing, Community analysis, Host-microbe interaction, Intestinal fungi, Microbiota, Microfungi, Mycobiome

## Abstract

Animal gut mycobiota, the community of fungi that reside within the gastrointestinal tract, make an important contribution to host health. Accordingly, there is an emerging interest to quantify the gut mycobiota of wild animals. However, many studies of wild animal gut mycobiota do not distinguish between the fungi that likely can reside within animal gastrointestinal tracts from the fungal taxa that are non-residents, such as macrofungi, lichens or plant symbionts/pathogens that can be ingested as part of the host’s diet. Confounding the non-resident and resident gut fungi may obscure attempts to identify processes associated with the authentic, resident gut mycobiota per se. To redress this problem, we propose some strategies to filter the taxa identified within an apparent gut mycobiota based on an assessment of host ecology and fungal traits. Consideration of the different sources and roles of fungi present within the gastrointestinal tract should facilitate a more precise understanding of the causes and consequences of variation in wild animal gut mycobiota composition.

## Gut microbiota impact host health

All animals host complex communities of commensal microbes (bacteria, fungi, protozoans, and viruses), the microbiota, which may comprise some hundreds to thousands of species [1–3]. Perhaps the most important, and certainly the most studied, component of host-associated microbiota are the communities of bacteria that inhabit the gastrointestinal tract (e.g. [1, 2, 4]). Numerous studies have identified diverse actions of gut bacteria that impact host health, for example provisioning of essential vitamins and metabolites by fermenting otherwise indigestible foodstuffs [1, 4], and helping to improve the host’s defence against pathogens through their dialogue with the host’s immune system [5–7].

An important feature of the digestive tract is that, as an open system, it will contain material from ingested microbes that may be dead, inactive, or form temporary interactions with the resident gut microbiota. Presence of this allochthonous microbial material provides an opportunity to identify spurious associations between the ‘apparent’ gut microbiota and properties of the host and its environment, potentially obscuring attempts to identify the core, resident gut microbiota and its functions. The impact of microbial material originating from dietary intake is a problem for studies of the microbiota of the stomach and small intestine that host relatively few (compared with the colon) living bacterial cells [8]. By contrast, as the human colon microbiota contains some 10^10^–10^11^ resident bacteria, the estimated relative abundance of ingested bacteria compared with the resident gut flora is some 0.0001–0.00001 fold [8]. Indeed, an analysis of the gut microbiota in human samples traced some bacteria to host diet, but the numbers of suspected dietary-associated material was low (towards the limit of detection) [2]. The direct contribution of dietary material to the apparent composition of gut bacteria is rarely examined, possibly because the hostile environment of the (vertebrate) stomach [9] is expected to kill many ingested microbes such that the composition of the gut microbiota should reflect the dynamics of the authentic resident bacteria. While it could be interesting to quantify the potential for ingested bacteria to pass alive through an animal’s digestive system in a greater diversity of host taxa, here we emphasise the general need to routinely assess the likely resident status of the gut fungal community, especially in studies of wild animals. The principal reasons to adopt this as standard practice for analyses of wild animal gut mycobiota are: (1) substantially fewer fungal cells reside within the animal gut than bacteria [10] and (2) many animals consume fungi. The outcome is an enhanced opportunity for a substantially reduced signal to noise ratio (i.e. a greater contribution of allochthonous material) in analyses of the gut mycobiota compared with analyses of gut bacteria.

## Gut mycobiota as an important but neglected component of animal microbiota

Although less well studied than the gut bacteria [10], there is increasing recognition that the gut mycobiota, the community of fungi that reside in gastrointestinal tracts, make an important contribution to host health [10–17]. Following such studies on humans and laboratory animals, there is emerging interest to establish the processes that determine the composition of the gut mycobiota of wild animals. For instance, host diet is associated with variation in the bat [18] and butterfly [19] gut mycobiota. Also, gut mycobiota of wild primates were species-specific and associated with habitat type, and possibly variation in diet [20].

An apparent feature of the gut microbiota is that there are orders of magnitude fewer fungal cells than bacterial cells in an animal gut microbiota. Estimates of total fungal load vary from 5 to 7% in laboratory mice faeces [21] to a perhaps more typical fraction of about 0.1% of the gut microbiota [1, 10]. The reduced abundance of fungal cells makes analyses of the gut mycobiota sensitive to the material from ingested fungi. For example, David et al.’s [2] analysis of the human gut microbiota identified an increase in the proportion of one food borne fungus (*Penicillium*) that apparently reduced the relative proportions of other fungi. The awareness that many humans regularly consume fungi as part of their diet, highlight the need to separate the resident gut mycobiota, which actively participates in host-microbiota interactions, from the transient ‘passengers’ who passively travel through the host’s gastrointestinal tract [14, 22, 23]. A meta-analysis of human gut mycobiota identified rather few fungal taxa (e.g. yeasts assigned to *Candida, Saccharomyces* and/or the Dipodascaceae, and *Malassezia* species) common to most studies, with many fungal taxa reported only once [24]. One interpretation of these data is that potentially many fungi identified in the human gut mycobiota studies are incapable of long-term residence in the gastrointestinal tract [22, 24]. In many instances, differentiation between the resident and ingested components of the gut mycobiota is aided in studies of human (and laboratory animal) microbiota where it is frequently possible to, for example, obtain detailed information about host lifestyle (e.g. diet), readily collect longitudinal data, and/or benefit from a large, collective research effort that is devoted to validating fungal species’ ability to reside within the gut (e.g. using culturing and experimentation). By contrast, studies of wild animal gut mycobiota are often based solely on the inference from an amplicon sequencing-based survey of a limited number of samples.

## Amplicon sequencing is an ‘unselective’ tool for analysis of gut mycobiota

One of the advances and drawbacks in analyses of microbial communities is the increasing use of high-throughput amplicon sequencing, also known as marker gene analysis [25] or metabarcoding [15, 26]. High-throughput amplicon sequencing relies on PCR to amplify a target region of DNA, which is typically part of the ribosomal RNA locus [15, 27], generating thousands to millions of sequence reads from samples containing DNA from diverse sources [15, 28]. Amplicons are compared against a reference database (e.g. [29]) to assign taxonomic identities to generated sequence variant (SV) groups (also known as operational taxonomic units (OTUs), phylotypes (PTs), etc.). Counts of the number of reads from each SV group can be further used to estimate the relative abundance of SVs [15, 30, 31]. High-throughput amplicon sequencing has revolutionised analyses of microbial communities by circumventing the need to isolate and culture microbes, a task that can be challenging and time consuming [16]. With the development of standardized protocols ([25]; https://earthmicrobiome.org/), extensive reference databases [29], and bioinformatics pipelines [15, 32], it is quite straightforward to use high-throughput amplicon sequencing to characterize microbial communities from diverse environments. A disadvantage associated with bypassing a culturing step to isolate and identify microbial species is that it can be hard to validate whether or not nominal species could reside within the environment from which their DNAs were recovered. Many analyses of host-associated gut mycobiota in wildlife overlook the issue that amplicon sequencing will amplify all fungal DNA present within a sample from an animal’s gut or faeces and the method does not distinguish between authentic resident gut fungi and other non-resident fungi.

## Separating the wheat from the chaff: non-resident fungi are present in analyses of wild animal ‘gut mycobiota’

**Fig. 1:**
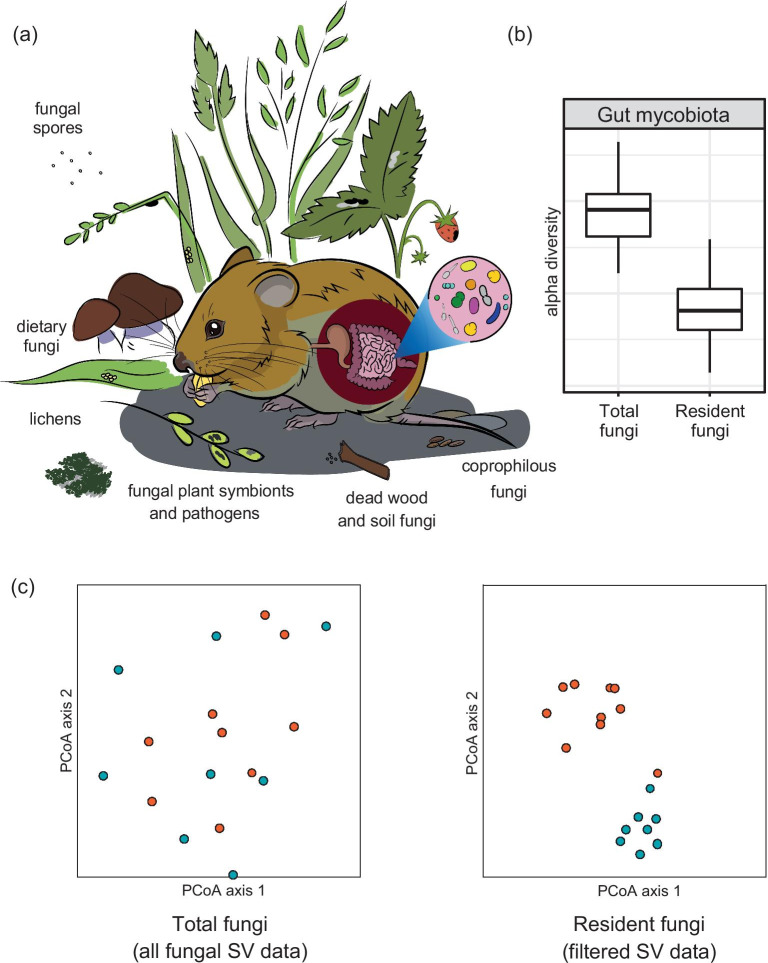
Potential sources of non-resident fungi in the gut mycobiota associated with wild animals. **a** Intake of non-resident fungi into an animal’s gastrointestinal tract can occur via *active* consumption, for example of dietary fungi or lichens, or via *passive* ingestion, for example of plant symbionts/pathogens, fungal spores from the environment, and/or by incidental ingestion of soil particles. **b** While inclusion of incidental fungi will elevate measures of alpha diversity, **c** the effect of inclusion of non-resident fungal sequence variant (SV) groups in analyses of beta diversity is unclear. In this hypothetical beta diversity analysis (e.g. principle coordinates analysis, PCoA) each point represents a single sample. Samples are coloured according to the host species (e.g. ‘orange’ and ‘blue’). Sample clustering pattern in the gut mycobiota associated with ‘orange’ and ‘blue’ hosts can be driven by a combination of the amount of environmental material ingested by the host and/or by variation in environmental fungal communities. Differences in sample grouping between the two ordinations illustrate the potential difficulties associated with inferring the likely causes of variation in wildlife gut mycobiota without identifying and filtering non-resident gut fungi

Animal gastrointestinal tracts may contain non-resident fungal material via diverse routes (Fig. 1), such as by active consumption or by incidentally ingesting commensal/symbiotic, pathogenic, coprophilous or other saprotrophic species (of fungi or spores) with their diet. Macroscopic fungi, lichens, and/or fungal plant pathogens or commensal fungi (for example ectomycorrhizal species) are likely components of the host diet rather than authentic, resident gut mycobiota. Numerous examples of inclusion of potential non-resident fungi in surveys of ‘gut mycobiota’ exist in the literature. For example, a study of two species of primates identified microfungal taxa in the Saccharomycetales to be dominant components of the primate gut mycobiota [20], but nonetheless reported some SV groups that were assigned to macrofungi (e.g. Polyporales, Agaricales) that include bracket-fungi and mushrooms, as well as some plant pathogens (e.g. members of *Pestalotiopsis* and *Lasiodiplodia*). Similarly, gut mycobiota in two species of wild mice were dominated by yeasts assigned to the genus *Kazachstania*, but contained SV groups assigned to a polypore, *Trametes versicolor* [33]. Further, SV groups assigned to macrofungi (*Steccherinaceae* and *Strophariaceae*, which include polyporoid and mushroom-forming species, respectively) form an abundant component of the apparent gut mycobiota of small rodents [34]. Indeed, SV groups assigned to macrofungi and plant pathogens or endophytes occur in analyses of gut mycobiota of diverse wild animal taxa, including macaques [35], bats [18], zebrafish [36], and birds [33, 37].

An appraisal of the likely resident or non-resident status of the SV groups present in a wild animal gut mycobiota is essential for a precise interpretation of how the gut fungal community composition may respond to environmental heterogeneity. For example, one response of the fungal community in fecal samples from yellow-necked mice inhabiting ecosystems that had experienced different levels of burning was a change in abundance of *Gelatoporia* sp. [34]. Since *Gelatoporia* is a genus of poroid crust fungi producing white rot that inhabits dead wood [38], these data may reflect the condition of the host’s environment and diet rather than an effect of environment on the resident gut mycobiota per se. More generally, it is relevant that the number of SV groups reported by studies of wild animal gut mycobiota varies considerably [20, 33, 39]. As the taxonomic diversity of an apparent ‘gut mycobiota’ can be inflated by the presence of fungal SVs from the host’s diet (Fig. 1 and Box 1), it is not possible to determine whether this variation in diversity reflects interspecific differences in the authentic, resident gut mycobiota or not. Inclusion of non-resident fungi in amplicon sequence data can interfere with producing reliable estimates of gut mycobiota alpha and beta diversity metrics, in an unpredictable manner due to the opportunistic nature of ingesting non-resident fungi (Fig. 1, see also Box 1).

Box 1 To illustrate the potential effect of filtering of sequence variant (SV) groups assigned to wild animal gut mycobiota we examined the data in Antwis et al. [34]. This study characterised the gut mycobiota of four species of rodent (bank vole, *Myodes glareolus n* = 152, wood mouse, *Apodemus sylvaticus n* = 24, striped field mouse, *A. agrarius n* = 25, and yellow-necked mouse, *A. flavicollis n* = 54) inhabiting the Chernobyl Exclusion Zone, Ukraine. Antwis et al. (2021) reported interspecific differences in the gut mycobiota community and some evidence that the proportion of two families of fungi associated with the host’s exposure to radionuclides. Briefly, read data were obtained from Genbank (accession number: PRJNA594002) and processed in qiime2 v.2020.6 [32], using the cutadapt [60] and dada2 [61] plugins to remove adapters and denoise the data. Taxonomy was assigned using vsearch [62] against the UNITE v.8 reference database [29]. To identify and filter likely non-resident SV groups (e.g. macrofungi, lichens) we classified fungal taxa based on their guild or growth forms using funguild v.1.2 [54], and also assigned broad categories of fungi as microfungi (or not) based on the information provided in the microfungi database (https://www.microfungi.org/Table1). Data were imported in r and merged into a phyloseq [63] object for analyses of alpha (SVs richness) and beta (Bray–Curtis dissimilarity) diversity. The *adonis2* function in r package vegan [64] was used to examine whether host species identity (e.g. bank vole, wood mouse, striped field mouse, yellow-necked mouse), sampling year (2017, 2018) and/or total absorbed dose rates of radiation (µGy/hr) explained variation in beta diversity. As a general assumption, we make a contrast between SVs representing potential dietary items (e.g. macrofungi, lichens) and against the remaining microfungal data (e.g. microfungi, yeasts, animal symbionts), but excluding those with an obvious relationship with plants (*e.g*. ectomycorrhizae, plant pathogens). For analyses, the data were rarefied to an even depth of 1500 (all data, and the filtered microfungi) or 500 (likely non-resident fungal SVs) reads per sample.

When considering all fungal SV data, these rodents apparently harbour a relatively diverse gut mycobiota with an average of 50–90 SVs per host species (Box Fig. 1a). Partitioning the data into likely resident and non-resident SVs results in a two-fold reduction of the estimates of the gut mycobiota diversity (Box Fig. 1a). Similarly, the variation in the gut mycobiota beta diversity between host species depends on the types of fungal SVs included (Box Fig. 1b). Indeed, there is somewhat clearer separation of the samples from bank voles and the three mouse species when all fungal SVs are included in the analysis compared with the analysis of only the probable resident gut fungi. Also, samples of wood mice tend to cluster with bank voles and differ from the samples of striped field and yellow-necked mouse when analysing likely non-resident fungal SVs. Moreover, filtering the data indicates that the strength of association between gut mycobiota and radiation dose rate can be affected by the types of fungal SVs included (*cf*. *R*^*2*^ and *p*-values in Box Table 1) and thus can influence the study conclusions. Hence, the data presented in Antwis et al. (2021) likely require careful filtering and re-analyses to make robust inferences about the possible ecological drivers of gut mycobiota variation. These data demonstrate a clear need for caution when analysing wildlife gut mycobiota communities characterised using amplicon sequence data alone.

## Strategies to identify non-resident and resident gut mycobiota SV groups

While classification of fungal taxa as likely residents and non-residents of the gut mycobiota is key for understanding processes that shape wild animal gut mycobiota communities, many studies of wildlife microbiota will lack the time and resources to make the necessary culturing experiments to confirm whether suspected members of the gut mycobiota could actually reside within the host’s digestive system. With this in mind, three broad strategies can be adopted to more accurately identify the likely causes and consequences of variation in wildlife gut mycobiota: (1) provide explicit data on host ecology, (2) improve experimental design and sampling, and (3) employ bioinformatic-based filtering of SVs (Table 1).

Host ecology and life stage play an important role in determining the expected proportion of non-resident fungi within the gut (Table 1). Many animals actively ingest fungi as part of their diet. For instance, omnivorous or herbivorous hosts are expected to ingest more non-resident fungi than insectivorous or carnivorous hosts (e.g. a comparison between phytophagous vs. insectivorous bats; [18]). While the timing and amount of fungi ingested is poorly documented for many species there is some evidence that active consumption of fungi by apparent herbivores could be quite common [40]. Greater diversity of non-resident fungi may be expected in samples from dietary generalists than dietary specialists. Indeed, analyses of gut mycobiota in butterflies [19], zebrafish [36], and captive tigers [41], have not uncovered SV groups belonging to obvious macrofungi or lichens, which is expected given the diets of these animal taxa. Nonetheless, these three studies have assigned SV groups to fungi inhabiting soil, or those that are plant pathogens or endophytes, whose membership in the resident gut mycobiota is unclear. Interestingly, the gut mycobiota of omnivorous yellow baboons was less diverse than that of the leaf-eating specialist red colobus, which were characterised by high levels of inter-individual variation with only few fungal SV groups shared among red colobus individuals [20]. As an example of ontogenetic shifts in gut mycobiota, humans experience a large reorganization of their gut mycobiota during the first year of life that accompanies the transition to solid food [16]. While there are no data on gut mycobiota establishment in early life for wild mammals, post-weaning individuals can be expected to ingest more non-resident fungi than neonatal individuals feeding on milk, thus host life stage can affect the intake of non-resident fungi into an animal’s gut and caution is needed. Thus, studies of gut mycobiota should include an explicit statement about the likely sources of ingested fungi, whether direct (e.g. macrofungi, lichens) or indirect (e.g. plant pathogens, symbionts or commensals), that would be detected quite readily by amplicon sequencing.

General guidelines to promote best practices in sampling, laboratory procedures and data analyses in studies of microbiota [25], and specifically mycobiota [15], have been presented elsewhere, and are not discussed in detail here—rather we highlight the need to further consider the ecologies of the hosts and fungi after implementing the best practice study design and analysis. Nonetheless, a robust analysis of the gut mycobiota of wildlife requires careful experimental design to focus the analysis on the authentic resident fungal taxa (Table 1). During the sampling step, collection of fresh fecal material is important to minimise the number of reads assigned to, for example, non-resident coprophilous fungi. Instant freezing will further decrease the chance of germination of any fungal spores present or overgrowth by moulds [15], more faithfully preserving the gut mycobiota community composition. Reagent and laboratory contamination can also impact analyses of gut mycobiota [42, 43]. Therefore, the use of negative controls (e.g. sample blanks) and/or positive controls with a mock community of known composition during DNA extraction, PCR and sequencing steps can help to detect potential contamination [15]. Working under a sterile laminar hood further decreases the risk of contamination with fungal spores from the built environment. In addition to these general guidelines, an important best practice to be adopted in analyses of gut mycobiota will be an assessment of the database of SVs to identify the taxa that, due to reasons described above, are unlikely to be resident members of the gut mycobiota.

Other sampling strategies to help identify the resident component of the gut mycobiota include source tracking [44] or making a time series analysis of the gut mycobiota composition (Table 1). Source tracking to identify the potential environmental source(s) of components of the host gut mycobiota could provide many insights into the processes that determine gut mycobiota community assembly and help identify the ‘gut specialist’ fungi; for example, butterfly [19] and caterpillar [45] gut mycobiota differ from the fungal communities associated with their potential food. Meaningful source tracking requires data from appropriate sources, such as those associated with potential diet and other appropriate features of host’s habitat, and obtaining these samples may be problematic (e.g. unknown diet, cryptic habitat use, etc.) and/or too resource intensive to acquire. Even with these challenges, it is possible to query one’s own or public (e.g. [46, 47]) data on environmental (e.g. soil, plant-associated) fungal communities to obtain some insight into the main groups of fungi that reside outside animal gastrointestinal tracts but within the host’s general living environment. We stress that this strategy would have to be applied with caution as some apparent environmental fungi could be transient themselves, for example incidentally introduced via animal faeces. Longitudinal sampling could be used to identify the stable (and likely resident) gut mycobiota, applying concepts of the common and temporal core mycobiota (e.g. taxa that occur in most individuals or sampling events within a defined host population or species) [48, 49]. Sampling humans at several time points revealed an apparently unstable gut mycobiota [22, 50, 51] with rather few fungal taxa detected across multiple samples due to the suspected presence of non-resident fungi. Longitudinal studies could also be useful to elucidate the effects of variation in diet and habitat use (Table 1). For instance, by sampling different seasons one could identify changes in the frequency of fungal fruiting bodies or plants (and associated fungal taxa) that constitute part of the host’s diet. Another method to identify the core gut mycobiota in a species of interest, is to compare fungal SV groups between wild and captive individuals, as wild animals are expected to ingest more non-resident fungi compared to their captive counterparts [36]. Collecting samples from diverse habitats can also help to define the core mycobiota (e.g. by applying frequency-based filtering) and thus reduce uncertainty when assigning authentic resident fungal taxa. Some caution would still need to be applied with any frequency-based filtering, as regular ingestion of plant-associated fungi (e.g. pathogens, symbionts or commensals) could identify certain fungal taxa as a ‘core species’ even if they cannot reside within the animal gut.

**Table 1:** Experimental design considerations and their relevance in studies of wild animal gut mycobiota. The table describes potential resources and strategies to identify and filter non-resident gut mycobiota in amplicon sequence data

Category	What features are important?	Why are these metadata important?
1. Metadata	Host diet	Host diet determines the likely amount of fungal contamination, e.g. omnivores should ingest more dietary fungi than carnivores
	Niche specialisation	Dietary specialisation determines the potential diversity of fungal contamination, e.g. generalists may ingest a higher diversity of environmental fungi than specialists
	Developmental stage	Host diet may change during development, e.g. adult mammals have a higher likelihood of ingesting fungi than neonates (that consume milk)
	Season	Both host diet and the type and availability of fungi can change with season
2. Sampling scheme	Longitudinal sampling	A time series analysis of the gut mycobiota can be used to define a set of core fungal sequence variant (SV) groups (especially in animals whose dietary fungi are seasonally available)
	Spatial sampling	Increasing the spatial extent of sampling can help to identify the core fungal SVs based on frequency of occurrence
	Time that sample is exposed to the environment	Fungi may rapidly colonise and grow on faeces after defecation (a more prominent risk for non-invasive sampling)
	Time since sample collection	Time between sample collection and preservation determines the potential for overgrowth of certain fungi
	Source tracking	Information about fungi that inhabit the host's environment and/or diet can be used to identify the taxa that have the potential to be commonly ingested
	Negative/positive controls	A diverse community of microbes can be present within the laboratory and the reagents, and contamination of samples by these microbes should be avoided as much as possible to maximise the signal to noise ratio in the data
	Sterile work	
3. Bioinformatic analyses	Positive filter using reference database(s), e.g. Targeted Host Fungi database	Fungal SVs that reside in the digestive systems of other animals are likely also to inhabit wild animal's gut. Note that reference databases are not always correct or up to date. The author(s) must clearly state the version of a database used in their analyses
	Filter SVs using fungal traits database(s), e.g. FUNGuild	Fungal SVs that have certain traits or growth forms, e.g. being 'macrofungi', are unlikely to be authentic residents of the gut microbiota
	Comparative analysis	Captive and/or laboratory animals often have less opportunities to ingest a diverse community of fungi, and these data may allow the likely ingested fungal SVs to be identified in wild conspecifics

**Box Fig. 1:**
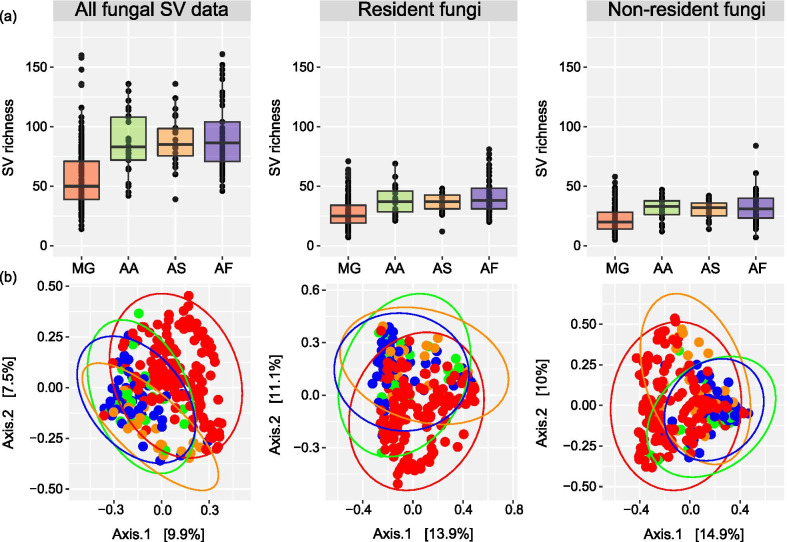
Interspecific variation in wild rodent gut mycobiota alpha and beta diversity. Measures of **a** alpha diversity based on the SV richness, and **b** beta diversity based on the Bray–Curtis dissimilarity for the gut mycobiota of four species of wild rodents (bank vole, *Myodes glareolus* MG, wood mouse, *Apodemus sylvaticus* AS, striped field mouse, *A. agrarius* AA, and yellow-necked mouse, *A. flavicollis* AF). The three data columns are faceted based on filtering of sequence variant (SV) groups, with data sets that include (1) all fungal SV data, (2) filtered SV data with only the likely resident or (3) non-resident gut fungi. **a** Box-and-whisker plots represent the median and interquartile range of SV richness. **b** Each point represents a single sample; samples are coloured according to the host species and are matched across the panels

**Box Table 1:** Statistical tests on the Bray–Curtis dissimilarity for the gut mycobiota of four species of wild rodents (bank vole, *Myodes glareolus*, wood mouse, *Apodemus sylvaticus*, striped field mouse, *A. agrarius*, and yellow-necked mouse, *A. flavicollis*). Statistical tests were performed using the *adonis2* function in r package vegan to (1) examine whether host species, sampling year and/or total absorbed dose rates of radiation (µGy/h) explained variation in beta diversity when all fungal sequence variant (SV) groups are included in the analysis, and (2) to compare explained variation with the analyses based on filtered SV data that either include only the likely resident or non-resident gut fungi

	All fungal SV data			Resident fungi			Non-resident fungi		
	*Df*	*R*^2^	*p*	*df*	*R*^2^	*p*	*df*	*R*^2^	*p*
Host	3	0.028	0.001	3	0.029	0.001	3	0.031	0.001
Dose	1	0.007	0.005	1	0.006	0.045	1	0.007	0.032
Year	1	0.019	0.001	1	0.021	0.001	1	0.016	0.001

Bioinformatic-based filtering can be used to focus the analyses on the authentic resident gut mycobiota. This strategy may be the only reasonable option when the sampling strategies mentioned above are not practical, for example because of a lack of information about host ecology or because it is not possible to obtain longitudinal samples. Some studies of gut mycobiota have filtered SV groups using a database, such as the Targeted Host Fungi database (THF; [52]); https://risccweb.csmc.edu/microbiome/thf/), as a positive filter of resident gut fungi [53]. However, even this manually curated reference database, which is optimized for annotation of gastrointestinal fungi of laboratory animals and humans, contains examples of likely non-resident macrofungi species, including *Agaricus bisporus* (a cultivated variety of the champignon mushroom) and *Boletellus projectellus* (a wild bolete fungus) (see THF v1.6.1, date accessed: January 15, 2021; [52]). As yet, there is no comparable database of fungi that are confirmed resident members of wild animal gastrointestinal tracts. Nonetheless, separating fungal SV groups into broad categories, such as microfungi, lichens, or macrofungi, is a useful first step as the latter two groups are very likely to be non-resident fungi. Although there is no universally accepted definition of macro- and microfungi, the Microfungal Collections Consortium (https://www.microfungi.org/Table1) summarizes known classes of microfungi. The software FUNGuild ([54]; http://funguild.org) provides an additional resource to identify and filter non-resident SV groups, for example, by classifying fungi as lichens (i.e. Guild = Lichenized) or by macrofungal morphological types (e.g. Growth Form = Agaricoid, Gasteroid, Polyporoid, Secotioid, etc.). Other databases for assigning functional traits and ecological information to fungal SVs include FUN^FUN^ [55] and FungalTraits [56]. While lichens and macrofungi perhaps represent reasonable lifestyles to categorize as non-residents of the animal gut, identifying the authentic resident gut microfungi will be challenging. Non-resident gut microfungi will likely reflect the dietary habits of the host and, for example, could include fungal taxa that are plant pathogens and plant symbionts (e.g. Guild = Plant Pathogen or Endophyte, respectively). To further complicate matters, some fungi are not easily defined ecologically [55]. Nonetheless, as an example, Ravenscraft et al. [19] conducted extensive filtering of fungal SVs based on fungal ecology and removed fungal SV groups assigned to wood decomposers and ectomycorrhizal, arbuscular, or ericoid mycorrhizal guilds from their butterfly gut mycobiota data. Using one or more of the databases of fungal traits, large amounts of SV data can be filtered as likely resident or non resident in the animal gut. It is important to emphasise the need for transparency and reproducibility when adopting this procedure, and thus the author(s) must clearly state the filtering steps used in their analyses and should provide (1) a comparison between the filtered data and all data (e.g. as supplemental information) and (2) accompanying metadata that lists (2.i) each SV and (2.ii) its assigned taxonomy, (2.iii) each SV’s guild (assigned by a defined version of a fungal traits database), and (2.iv) the resident/non-resident status assigned by the author(s).

## Don’t ‘throw the baby out with the bathwater’: need for caution when filtering fungal SV data

Although we advocate that informed filtering of the fungal SVs identified in a gut or faecal sample is required to better characterise the likely resident gut mycobiota of wild animals, we stress that the filtering process yields an informed ‘hypothesis’ about some aspect of the wild animal gut mycobiota. In most studies there will be a need to further consider possible roles of the different classes of SVs that exist within the data. For example, ingested probiotic bacteria may elicit functional changes in the gut microbiota even if the probiotic bacteria have little detectable impact on the microbiota community per se [57]. Focusing an analysis only on the suspected resident gut mycobiota could also overlook the possible functions that transient fungal SVs could perform during their passage through the host’s gastrointestinal tract. Moreover, an uncritical removal of fungal SVs based on their traits could hinder the identification of novel taxa involved in authentic fungal-animal relationships. For instance, SVs assigned to ‘cyanobacteria’ have been previously identified in samples of animal microbiota. At first glance, such data were often thought to represent non-resident bacteria, possibly as a contaminant fraction, as photosynthetic cyanobacteria should not reside in animal gastrointestinal tracts. However, subsequent metagenomic analyses uncovered the genomes of a novel phylum, Melainabacteria, an apparent sibling phyla to the Cyanobacteria that can perform potentially useful metabolic services for its animal host [58]. In this case, dismissing SVs based on putative traits could have resulted in overlooking this novel and unique phylum of resident-microbiota. With this in mind, the value of maintaining explicit metadata about the putative association of each SV with its host (e.g. resident, dietary, core, temporary) would provide a valuable resource to stimulate discussion and to better enable other researchers to collate datasets and examine the occurrence of SVs associated with hypothesised resident, transient, dietary, etc. status within wildlife gut mycobiota.

## Concluding remarks

Quantifying the diversity and function of the gut mycobiota is likely to provide a better understanding of processes that affect health and fitness in wild animals. Frequent occurrence of dietary material and/or notable plant symbionts and plant pathogens in wild animal gut mycobiota communities highlights the need for clarity about the definition of an authentic gut mycobiota as identified in typical high-throughput amplicon sequencing surveys. While best practices for technically sound microbiota/mycobiota analyses are covered elsewhere [15, 25], there is a clear need to consider more deeply the life history and ecology of the Fungi when analysing gut mycobiota of wildlife. Hence, here we propose a checklist to accompany analyses of wild animal gut mycobiota, specifically focusing on the strategies and available resources to identify and filter non-resident gut mycobiota (Table 1). Before data on wild animal gut mycobiota accumulate, it is essential that studies include an explicit step of ‘informed filtering’ about which SVs reflect the resident and non-resident components of the gut mycobiota. It would be helpful to adopt a standard practice whereby each manuscript is accompanied by metadata that contains an annotated list of fungal SV groups, their abundance, prevalence among examined individuals, putative classifications (e.g. as macrofungi, microfungi or lichens, mushrooms, polypores, etc.), and representative sequences. Such metadata should be deposited as standard supplementary information and would facilitate meaningful re-use of data and meta-analyses. Without a reasonable attempt to understand the sources and ecology of fungal SV groups identified from animal gut/fecal samples, analyses of the diversity and function of wild animal ‘gut mycobiota’ could be compromised (Box 1). While fungi have diverse life histories and ecologies, which are poorly characterized for many species, it is still better to adopt some informed filtering for SV groups that likely do not reside in the animal gut than to overlook this issue. Moreover, recognizing the diversity of fungal SV groups that can be retrieved from gut and fecal samples of wild animals may provide new research opportunities such as using animals as ‘natural samplers’ of environmental fungal diversity or to track fungal pathogens [59]. While there are many opportunities to identify the interactions between wild animals and their associated fungal communities, there is a current critical need for more consideration of what constitutes resident versus non-resident gut mycobiota.

## Data Availability

The raw sequence data originally presented in the study by Antwis et al. 2021 (https://doi.org/10.1111/1365-2656.13507) are available from the NCBI SRA database under accession number PRJNA594002. All the other data generated or analysed during this study are included in this published article (and its supplementary information files).
